# Hippocampal Subfield Volumes in First Episode and Chronic Schizophrenia

**DOI:** 10.1371/journal.pone.0117785

**Published:** 2015-02-06

**Authors:** Mitsuhiko Kawano, Ken Sawada, Shinji Shimodera, Yasuhiro Ogawa, Shinji Kariya, Donna J. Lang, Shimpei Inoue, William G. Honer

**Affiliations:** 1 Department of Neuropsychiatry, Kochi Medical School, Kochi, Japan; 2 Department of Psychiatry, Aki General Hospital, Kochi, Japan; 3 Department of Radiology, Hyogo Prefectural Kakogawa Hospital, Hyogo, Japan; 4 Departments of Diagnostic Radiology and Radiation Oncology, Kochi Medical School, Kochi, Japan; 5 Department of Radiology, University of British Columbia, Vancouver, Canada; 6 Department of Neuropsychiatry, Aizu Medical Center, Fukushima Medical University, Fukushima, Japan; 7 Department of Psychiatry, University of British Columbia, Vancouver, Canada; University of California, San Francisco, UNITED STATES

## Abstract

**Background:**

Reduced hippocampal volume in schizophrenia is a well-replicated finding. New imaging techniques allow delineation of hippocampal subfield volumes. Studies including predominantly chronic patients demonstrate differences between subfields in sensitivity to illness, and in associations with clinical features. We carried out a cross-sectional and longitudinal study of first episode, sub-chronic, and chronic patients, using an imaging strategy that allows for the assessment of multiple hippocampal subfields.

**Methods:**

Hippocampal subfield volumes were measured in 34 patients with schizophrenia (19 first episode, 6 sub-chronic, 9 chronic) and 15 healthy comparison participants. A subset of 10 first episode and 12 healthy participants were rescanned after six months.

**Results:**

Total left hippocampal volume was smaller in sub-chronic (*p* = 0.04, effect size 1.12) and chronic (*p* = 0.009, effect size 1.42) patients compared with healthy volunteers. The CA2-3 subfield volume of chronic patients was significantly decreased (*p* = 0.009, effect size 1.42) compared to healthy volunteers. The CA4-DG volume was significantly reduced in all three patient groups compared to healthy group (all *p* < 0.005). The two affected subfield volumes were inversely correlated with severity of negative symptoms (*p* < 0.05). There was a small, but statistically significant decline in left CA4-DG volume over the first six months of illness (*p* = 0.01).

**Conclusions:**

Imaging strategies defining the subfields of the hippocampus may be informative in linking symptoms and structural abnormalities, and in understanding more about progression during the early phases of illness in schizophrenia.

## Introduction

Reduced hippocampal volumes are a more prominent morphological feature of chronic schizophrenia in comparison to the more subtle volumetric changes seen in first episode or early schizophrenia patient groups [1–3]. The hippocampus consists of major subfields, including the Cornus Ammonis (CA)1, CA2-3, dentate gyrus (DG), presubiculum, and subiculum. The perforant pathway input from the entorhinal cortex induces serial excitatory transmission through DG, CA2-3, CA1, and back to the entorhinal cortex through the subiculum. This trisynaptic pathway has been considered to be the fundamental network closely linked to learning and memory [2]. Several papers in hippocampal morphology in schizophrenia have indicated that pathological alterations were evident in subfield or hemisphere specific manner [4–6]. Although the significance of structural changes in hippocampal subfields was proposed, it remained uncertain whether the subfields would be globally reduced or specific parts of subfields such as CA1, CA3, DG or subiculum are subject to more localized change.

Recently, more detailed analyses of hippocampal shape and surface morphometry have revealed both localized and lateralized findings [7–11], including subregional differences into CA1 and CA2 subfields in first episode patients [9]. A multimodal imaging strategy indicated that deformations in the anterior hippocampus were related to cortical thinning, and the degree of disruption in structural hippocampal-cortical connectivity was associated with severity of negative symptoms [10]. Progressive changes in hippocampal shape were also reported [12]. Using a three-dimensional surface mapping technique, the extent of “deflation” of the hippocampus in schizophrenia was associated with duration of illness in a sample of 67 patients with mean age 39 years and duration of illness 13 years [13]. Severity of both positive and negative symptoms was also associated with this measure of change in hippocampal structure. The finding of an association between surface shape abnormalities referable to the CA1 subfield and positive symptoms was also observed in a study of patients mean age 34 and duration of illness 10 years [14].

Current high-resolution image segmentation strategies now provide both the opportunity for surface mapping, as well as the opportunity to obtain hippocampal subfields volumes from magnetic resonance images [15]. These newer imaging strategies allow for the assessment of subfields including CA4 and the dentate gyrus that may not be readily detected by surface analysis techniques. Recently, a large study including chronic schizophrenia, schizoaffective disorder, and bipolar disorder with psychosis reported smaller total hippocampal volume across all diagnostic groups compared to healthy volunteers, with the most prominent reductions seen in the schizophrenia group within the CA2-3, CA4-DG and the subicular subfields [16]. In this study smaller volumes were associated with more severe positive symptoms, and greater cognitive impairment, but not with antipsychotic treatment. A second study of hippocampal subfield volumes in 21 multi-episode chronic patients reported an association between CA1 and CA2-3 volumes and positive symptoms [17]. Negative symptoms were not associated with subfield volumes in this study, and comparisons to subfield volumes in healthy participants was not reported. Finally, a study of young family members of probands with schizophrenia reported smaller subiculum volumes compared with healthy controls with no family history [18].

Studies of first episode patients, with minimal medication treatment, and longitudinal studies are required to determine if specific subfields are affected early in the course of illness, and if progressive changes occur. Similar strategies to investigate cognitive impairment in the elderly have suggested that initial volume loss in CA1 was associated with mild cognitive impairment, followed by volume loss across multiple subfields and transition to Alzheimer’s disease [19]. While cognitive deficits are an established characteristic of schizophrenia, whether the relationships between hippocampal subfield and cognitive deficits are similar in schizophrenia compared to Alzheimer’s patients are unknown.

For the current investigation we hypothesized that the volumes of hippocampal subfields CA1, CA2-3, DG, presubiculum, or subiculum would be smaller in chronic schizophrenia compared with healthy participants, and that there would progressively larger differences related to duration of illness. Furthermore, we expected the degree of volume change to be correlated with the severity of psychopathology. We used a high-resolution segmentation strategy based on a recently developed automated technique (see [15]) to examine hippocampal subfields in groups of first episode, sub-chronic and chronic patients in comparison with healthy participants. As well, to further examine the possibility of progression, we performed a second scan six months following baseline in a subset of first episode patients and healthy comparison participants.

## Methods

### Participants

Thirty-four ethnically Japanese patients were recruited from in- or outpatient services of Kochi Medical School, Hosogi Unity Hospital, and Tosa Hospital in Kochi Prefecture, Japan. Demographic data and clinical features of illness appear in Table 1. Nineteen of the patients were in their first episode of illness at baseline, defined as having made their first contact for treatment of psychotic symptoms, and having less than one month of lifetime antipsychotic treatment. Patients who were ill for six months to five years were categorized as sub-chronic (N = 6),) and who were ill for over five years as chronic (N = 9). Exclusion criteria included a history of head injury with loss of consciousness, other neurological disorder, current substance abuse or dependence. Participants had no clinically significant brain pathology, as determined by a neuroradiologist’s review of the MRI scans. The diagnosis of schizophrenia was made according to ICD-10 criteria. Socio-economic status (SES) was analyzed based on educational years [20].Clinical assessments included the Positive and Negative Syndrome Scale (PANSS) [21], the Clinical Global Impression (CGI) [22], and Global Assessment of Functioning (GAF) (DSM-IV-TR). The Schizophrenia Cognition Rating Scale (SCoRS), an 18 item scale of real world cognitive deficits, was used to evaluate cognitive functioning. The global rating of SCoRS was analyzed in retrograde fashion [23,24]. Chlorpromazine dose equivalents were calculated for all antipsychotic drugs [25–27]. First episode, sub-chronic and chronic patients were scanned at baseline. Ten first episode patients were rescanned after six months (mean 192 days, SD 16). First episode patients were taking stable doses of antipsychotic medication (risperidone n = 6, olanzapine n = 3, perospirone n = 1; mean chlorpromazine equivalents = 300 mg) at the time of the follow-up scan. Fifteen healthy volunteers were recruited from the neighboring region. Healthy controls had no past or current history of mental illness, and met the same exclusion criteria as patients. Twelve healthy volunteers were rescanned after six months (mean 186 days, SD 15).

**Table 1:** Demographic and clinical characteristics of participants at baseline, mean (standard deviation).

	Healthy Controls		Schizophrenia					
	(N = 15)		FES (N = 19)		SCS (N = 6)		CS (N = 9)	
Age (yrs) ^a^	25.0	(4.6)	25.1	(6.8)	22.2	(3.7)	36.8	(6.7)
SES^b^	5.7	(0.9)	4.5	(1.2)	4.2	(1.6)	4.0	(1.0)
Parental SES	4.9	(1.0)	4.6	(1.1)	4.3	(0.8)	4.3	(1.2)
SCoRS			4.0	(1.3)	5.2	(2.3)	5.4	(1.8)
Handedness (right, left)	15, 0		15, 4		5, 1		9, 0	
Sex (M, F)	10, 5		9, 10		3, 3		6, 3	
Chlorpromazine-Equivalent dose at scan (mg/day) ^c^			263	(156)	758	(218)	781	(116)
PANSS score								
Positive subscale			16.8	(3.1)	16.7	(6.1)	19	(5.1)
Negative subscale ^d^			14.6	(4.4)	19	(4.9)	18.1	(2.8)
General subscale			30.1	(5.4)	32.7	(4.9)	35.8	(6.1)
Total score			61.5	(10.3)	68.3	(14.7)	72.9	(12.0)
CGI			4.3	(0.7)	4.2	(1.2)	4.4	(0.9)
GAF			45.8	(9.5)	50	(16.7)	44.8	(12.7)

SES: socio-economic status, SCoRS: Schizophrenia cognition rating scale, PANSS: Positive and Negative Syndrome Scale, CGI: Clinical global impression, GAF: Global assessment of function, FES: first episode schizophrenia, SCS: sub-chronic schizophrenia, CS: chronic schizophrenia

^a^ Overall difference between groups F = 10.9, *p* < 0.001

^b^ Overall difference between groups F = 5.36, *p* = 0.003

^c^ Different between groups F = 11.9, *p* < 0.001

^d^ Different between groups F = 3.78, *p* = 0.03

### Ethical considerations

Ethical review and approval for this study was provided by the Kochi Medical School ethical committee. Subjects were recruited from two streams of independent participants. For subjects enrolled in our stand-alone longitudinal study all subjects directly provided written informed consent for our specific study. Additional subjects ascertained from part of a separate cross-sectional study provided broad-based informed written consent to have their medical records and charts included in any institution-based research projects, such as the current study. Those whose data were included from the cross-sectional pool were given notification of the inclusion of their medical records via the medical school's webpage interface, as stipulated by the clinical ethics committee. In cases where the participants may have had a compromised capacity or ability to give consent, next of kin, care takers or guardians consented on their behalf.

### Image acquisition and processing

**Fig 1:**
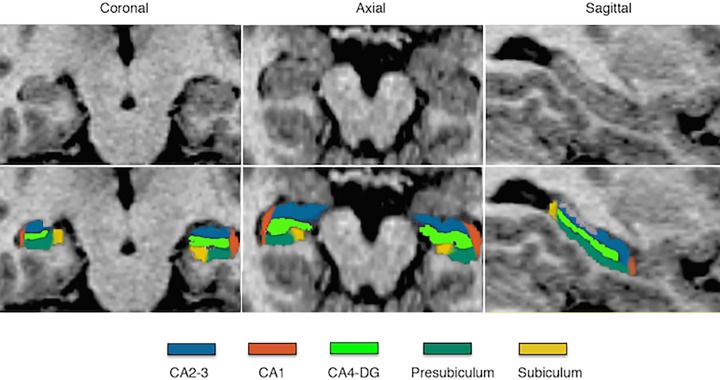
Hippocampal subfield segmentation in a representative subject.

All participants were examined on 1.5 T GE scanner at Kochi Medical School using a 3D T1-weighted SPGR sequence (TE = 4 ms; TR = 9.7 ms; flip angle = 12°; matrix = 256 × 256; FOV = 200 mm; slice thickness = 1.5 mm with no gap; 0.78 × 0.78 × 1.5 mm voxels; 124 slices). Total intracranial and hippocampal volumes were determined using the FreeSurfer software package version 5.1 (http://surfer.nmr.mgh.harvard.edu/fswiki). The hippocampus segmentation was fully automated without manual editing. All automated outputs were visually inspected to ensure there were no technical failures or mislabeling. Two subjects were excluded after visual examination due to failure of the segmentation algorithm. The FreeSurfer definition of the hippocampal subfields includes the dentate gyrus (CA4-DG), the Ammon’s horn subfields (CA1, CA2-3), the subiculum and presubiculum [15]. The hippocampal subfield delineations are illustrated in Fig. 1. The delineated images were similar to the previous results processed with FreeSurfer [15,17,19,28]. The mean subfield volumes we obtained are very similar to those reported by the algorithm developers [15]. As well, we calculated the intraclass correlation coefficient for n = 12 scans of healthy participants performed six months apart. The ICC for the left hippocampus total volume was 0.90, and for the right 0.86. For the 10 left and right hippocampal subfields, the mean ICC value was 0.88, with a range of 0.71 for the right CA1 subfield, to 0.95 for the left CA2-3. For longitudinal analyses, we used FreeSurfer’s longitudinal stream to process two serial MRIs from study participants to provide accurate estimates of subtle changes over time [29].

### Statistical analyses

To test the main hypotheses concerning total hippocampal and subfield volumes at baseline, analysis of variance (ANOVA) was performed with group (first episode schizophrenia, sub- or chronic schizophrenia, control) entered as a main effect, and age and intracranial volume entered as covariates. Whole left and right hippocampal volumes were tested first, with a *p*-value set at 0.025 for statistical significance. A difference in total volume between groups was followed up by subsequent statistical comparisons of the five subfield volumes, with a *p*-value set at 0.01 for statistical significance. For post-hoc comparisons of total or subfield volumes between the three diagnostic groups, t-tests were used with the Hochberg approach to correct for multiple testing, as previously used in other MRI studies [16,30,31]. Repeated measures ANOVA was performed on in hippocampal subfield volume in the first episode group and in healthy volunteers for those who had follow-up imaging with group as a between-subjects factor, and time (baseline, follow-up) as a within-subjects repeated measure.

Demographic and clinical variables were analyzed with chi-square or ANOVA as appropriate. Relationships between hippocampal total and subfield volumes, demographic and clinical assessments (duration of illness, age, positive and negative symptoms) were explored with parametric or non-parametric techniques depending on the distribution properties of the variables. Analyses were conducted using SPSS version 22.0 (SPSS Tokyo, Japan) or JMP 10 (SAS Institute, Cary NC, USA).

## Results

### Demographic and clinical data of participants

Demographic and clinical variables in the cross-sectional study are presented in Table 1. ANOVA of the four groups revealed significant differences in age (F = 10.9, *p* = 0.036). Post hoc analyses revealed that the mean age of chronic group was significantly older than other groups. Although there were significant differences in scores of SES between the groups (F = 5.4, *p* = 0.003), we did not find differences in parental SES. Chlorpromazine-equivalent doses at scan in sub-chronic and chronic group were higher than first episode group. Scores for negative symptom severity were higher in the sub-chronic and chronic groups than in the first-episode group. SCoRS, CGI, and GAF did not show significant differences between groups. In the longitudinal study, the first episode took a mean dose of 290 mg chlorpromazine equivalent antipsychotics for about 15 days before the first scan. Controls had higher SES score than first episode group, but there was no difference in parental SES between the groups. There was no difference in age, sex, or intracranial volume between the groups. First episode subjects at follow-up were significantly improved in total score (F = 5.65, *p* = 0.041) and positive symptom score (F = 9.41, *p* = 0.013) in PANSS, CGI (F = 21.00, *p* = 0.001), and GAF (F = 17.80, *p* = 0.002).

### Hippocampal total and subfield volumes at baseline

**Fig 2:**
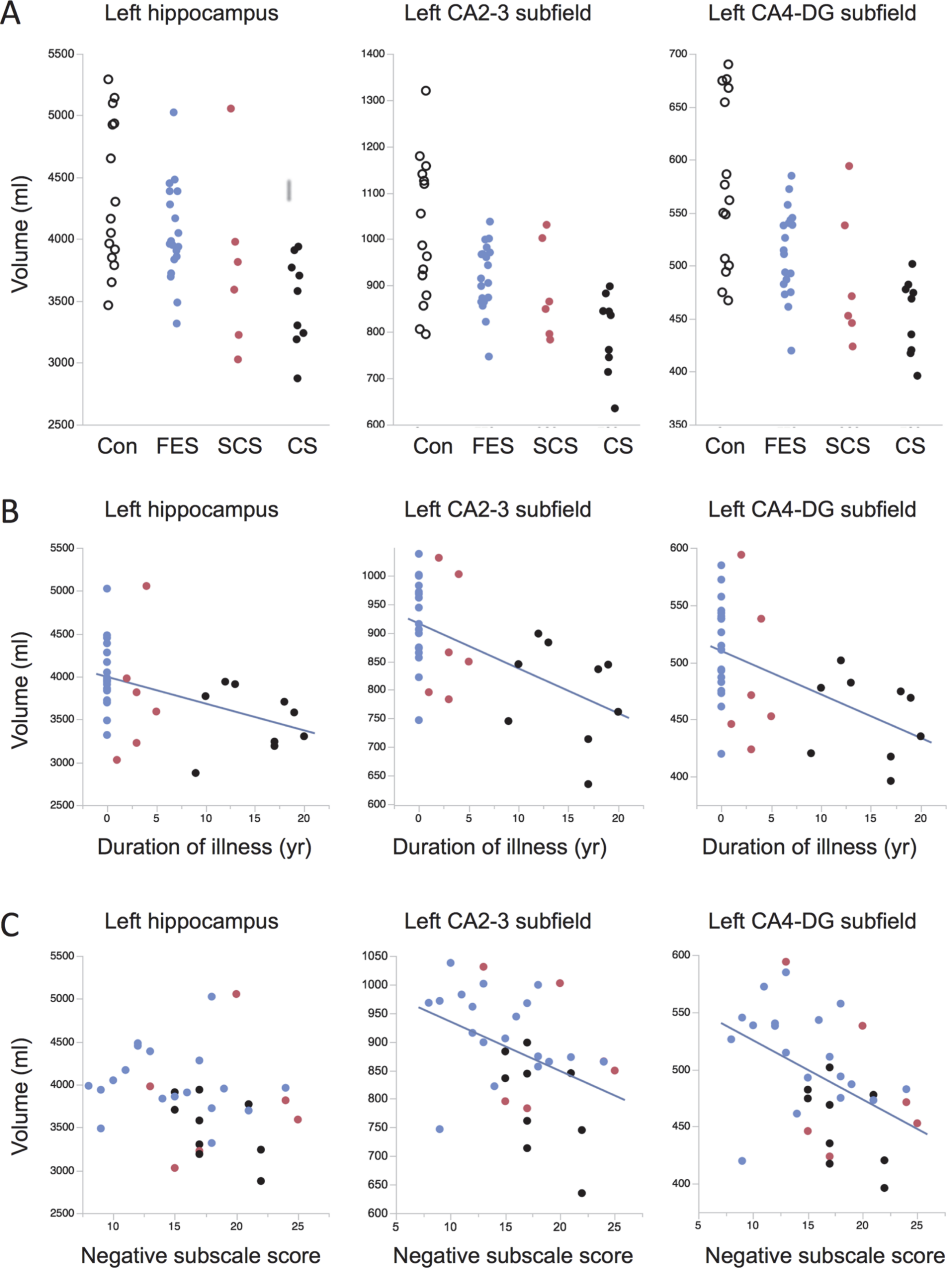
Left hippocampal total and subfield volumes in control and schizophrenia groups. **(A)** Hippocampal total and subfield volumes in healthy controls (Con, black open circles), first episode schizophrenia (FES, blue), sub-chronic schizophrenia (SCS, red) and chronic schizophrenia (CS, black) participants. Mean volumes were smaller in CS than Con for total (*p* = 0.004), CA2-3 (*p* = 0.003) and CA4-dentate (DG) (*p* < 0.001) subfields. (**B)** Relationships between duration of illness and hippocampal volumes. Non-parametric correlations were statistically significant for total (*p* = 0.004), CA2-3 (*p* = 0.003) and CA4-DG (*p* = 0.002). (**C)** Relationships between negative subscale scores on the PANSS and hippocampal volumes. Parametric correlations were statistically significant for CA2-3 (*p* = 0.02) and CA4-DG (*p* = 0.007).

Total hippocampal volume on the left was significantly different between groups, with both healthy and first episode schizophrenia participant volumes larger than chronic schizophrenia (Table 2). In sub-chronic schizophrenia the mean volume of the left hippocampus was 12.6% smaller than controls (*p* = 0.04, effect size 1.12); in chronic schizophrenia, the mean volume was 17.0% smaller than controls (*p* = 0.009, effect size 1.42) (Fig. 2).

**Table 2:** Total and subfield volumes (ml) for the hippocampus at baseline.

	Healthy (N = 15)		FES (N = 19)		SCS (N = 6)		CS (N = 9)		Healthy vs FES		Healthy vs SCS		Healthy vs CS	

	LSM	(SEM)							p-value	Cohen d	p-value	Cohen d	p-value	Cohen d
Left hippocampus^a^	4270	(111)	4078	(92)	3800	(166)	3607	(169)	0.19	0.46	0.04	1.12	0.009	1.42
CA1	345	(10)	316	(8)	319	(15)	305	(16)						
CA2-3^b^	1000	(26)	921	(22)	883	(39)	834	(40)	0.03	0.8	0.03	1.19	0.006	1.5
CA4-DG^c^	571	(14)	517	(11)	491	(21)	460	(21)	0.004	1.06	0.004	1.51	0.0003	1.89
Presubiculum	466	(16)	468	(13)	461	(24)	454	(24)						
Subiculum	630	(20)	606	(16)	581	(30)	543	(30)						
Right hippocampus	3943	(116)	4118	(96)	4048	(174)	3997	(176)						
CA1	333	(10)	326	(8)	351	(15)	345	(15)						
CA2-3	922	(26)	941	(21)	971	(39)	941	(39)						
CA4-DG	522	(15)	525	(12)	542	(22)	533	(23)						
Presubiculum	481	(17)	470	(14)	437	(25)	432	(25)						
Subiculum	618	(18)	606	(15)	598	(27)	599	(28)						

Hippocampal volumes are corrected for age, sex and intracranial volume. LSM: least squares mean, SEM: standard error of the mean. Post-hoc p-values are corrected for three comparisons. FES: first episode schizophrenia, SCS: sub-chronic schizophrenia, CS: chronic schizophrenia

^a^Difference between groups F = 4.51, *p* = 0.008

^b^Difference between groups F = 4.87, *p* = 0.005

^c^Difference between groups F = 8.14, *p* = 0.0002

The hippocampal subfields showed distinctive patterns of differences between groups. Volumes of the presubiculum and CA1 subfields were relatively unchanged, and although the subiculum was smaller in chronic patients, there was no statistically significant difference between groups. In contrast, the CA2-3 region was smaller in sub-chronic (*p* = 0.03, effect size 1.19) and chronic patients (*p* = 0.006, effect size 1.50) than healthy volunteers, with first episode patients having intermediate volumes that were not different from either group. The volume of the CA4-DG subfield was significantly smaller in first episode (*p* = 0.004, effect size 1.06), sub-chronic (*p* = 0.004, effect size 1.51) and chronic groups (*p* = 0.0003, effect size 1.89) compared to healthy volunteers.

### Associations with clinical features of illness

There were no statistically significant correlations between age and total hippocampal or age and subfield volumes on either side. We conducted exploratory analyses of associations between duration of illness and volumes, using Kendall’s tau as the measure since the data was not normally distributed, and because there were multiple zero values for duration of illness when the first episode participants were included. As seen in Fig. 2, statistically significant associations were observed for total left hippocampus (Kendall’s tau = -0.38, *p* = 0.004), as well as left CA2-3 (Kendall’s tau = -0.39, *p* = 0.003) and CA4-DG (Kendall’s tau = -0.40, *p* = 0.002). These findings must be interpreted cautiously, as removal of the first episode participants decreased the strength of the association below the level of statistical significance.

Severity of positive symptoms did not correlate with the three target hippocampal subfield volumes. Severity of negative symptoms was correlated with left CA2-3 (r^2^ = 0.16, *p* = 0.02) and with left CA4-DG (r^2^ = 0.21, *p* = 0.007), but not with total left hippocampal volume (Fig. 2). Effects of negative symptom severity remained statistically significant when age, sex, and intracranial volume were added to the model. There were no statistically significant correlations between hippocampal volumes and antipsychotic doses.

### Changes in hippocampal total and subfield volumes over the first six months of treatment

**Fig 3:**
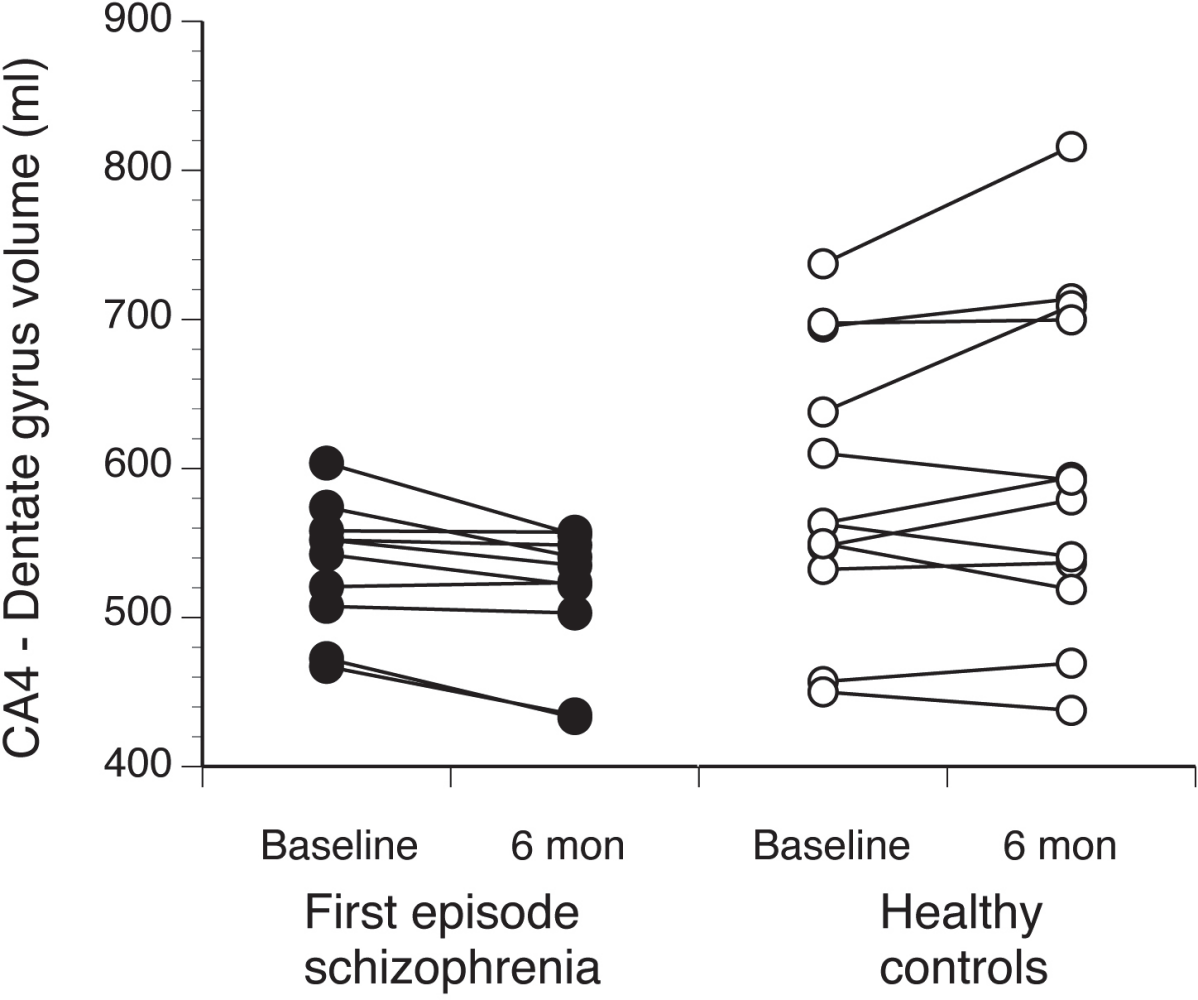
Baseline and follow-up volumes of left hippocampus CA4-dentate gyrus subfield in first episode schizophrenia and healthy control participants. A statistically significant diagnosis-by-time interaction was observed (*p* = 0.01).

At follow-up, the severity of total and positive symptoms was reduced in the first episode schizophrenia patients (see Table 3). Total hippocampal volumes did not differ over time between healthy volunteers and patients with first episode schizophrenia. The left CA4-dentate gyrus subfield showed a statistically significant group-by-time interaction (*p* = 0.01). Volumes increased slightly in healthy volunteers over six months, and declined slightly in first episode schizophrenia (Fig. 3 and Table 4).

**Table 3:** Demographic and clinical characteristics of participants in the longitudinal study, mean (standard deviation).

	Healthy Controls		First episode schizophrenia			
	(N = 12)		(N = 10)			
			Baseline		Six months	
Age (yrs)	25.4	(4.8)	25.8	(5.6)		
SES ^a^	5.7	(0.7)	4.7	(1.3)		
Parental SES	4.5	(0.9)	4.3	(1.2)		
SCoRS	3.5	(1.1)	3.5	(0.9)		
Handednes (right,left) ^b^	(12, 0)		(8, 2)			
Sex (M, F)	(5, 7)		(4, 6)			
Chlorpromazine-Equivalent dose at scan (mg/day)			290.0	(192)	310.0	(145)
PANSS score						
Positive subscale ^c^			17.3	(2.8)	11.5	(5.6)
Negative subscale			13.6	(5.0)	11.9	(2.5)
General subscale			30.8	(5.7)	26.2	(7.4)
Total score ^c^			61.7	(11.1)	49.6	(13.6)
CGI ^d^			4.3	(0.8)	2.9	(1.1)
GAF ^d^			43.3	(9.7)	63.8	(16.7)

Antipsychotics used were: risperidone (N = 6), olanzapine (N = 3), perospirone (N = 1). SES: socio-economic status, SCoRS: Schizophrenia Cognition Rating Scale, PANSS: Positive and Negative Syndrome Scale, CGI: Clinical global impression, GAF: Global assessment of function.

^a^ Overall difference between groups F = 4.90, *p* = 0.04

^b^ Different between groups Chi-square = 4.17, *p* < 0.05

^c^ Different over time, F = 5.65–9.41, *p* < 0.05

^d^ Different over time, F = 17.80–21.00, *p* < 0.005

**Table 4:** Total and subfield volumes (ml) of participants in the longitudinal study, mean (standard deviation).

	Healthy Controls					First episode schizophrenia			
	(N = 12)					(N = 10)			
	Baseline		Six months			Baseline		Six months	
Left-Hippocampus	4481	(673)	4513	(722)		4248	(450)	4146	(556)
CA1	339	(56)	346	(58)		324	(25)	311	(29)
CA2-CA3	1036	(181)	1044	(186)		936	(77)	911	(89)
CA4-DG ^a^	587	(92)	601	(113)		535	(43)	515	(46)
Presubiculum	483	(74)	479	(71)		458	(49)	456	(47)
Subiculum ^b^	639	(106)	663	(111)		604	(43)	595	(52)
Right-Hippocampus	4291	(444)	4455	(706)		4138	(343)	4107	(379)
CA1	344	(54)	357	(64)		313	(23)	312	(20)
CA2-CA3	1014	(178)	1003	(150)		906	(70)	911	(54)
CA4-DG	574	(100)	575	(96)		508	(36)	512	(30)
Presubiculum	511	(85)	490	(63)		460	(53)	462	(61)
Subiculum	654	(85)	665	(89)		608	(45)	597	(49)

^a^ Group x time interaction F = 7.62, *p* = 0.01

^b^ Group x time interaction F = 4.76, *p* = 0.04

## Discussion

In this study, the CA2-3 and CA4-DG subfields of the left hippocampus were more prominently reduced than the CA1 region, or the presubiculum/subiculum in patients with schizophrenia. The graded differences in subfield volumes between health volunteers, first episode, and chronic schizophrenia, and the observation of increasing loss of volume in the CA4-DG subfield during the first six months following presentation for care, suggest that a dynamic process related to illness or treatment occurring. In the overall patient group more severe negative symptoms were associated with smaller subfield volumes.

The affected hippocampal subfields in this patient group are similar to those reported in postmortem studies to have neurochemical and synaptic changes associated with schizophrenia [2,4–6,32,33]. The relative predominance of findings on the left side, and the sparing of the CA1 region was also consistent with postmortem studies of the hippocampus in schizophrenia [5]. This is in contrast to the pathology of Alzheimer’s disease, where the CA1 region is affected early in the course of illness.

Of interest, abnormalities of the left hippocampus are more frequently observed schizophrenia [5]. Moreover, traumatic stress or abusive events in childhood have a propensity to contribute to smaller left hippocampal volumes in adulthood [34–37]. In addition to the preferential involvement of left hippocampus in schizophrenia, the asymmetric hippocampal changes in the current study may hsve been influenced by psychosocial stress or mood change. At baseline in sub-chronic and chronic subjects, CA2-3, and DG volumes were decreased compared to healthy volunteers as well as left total hippocampus, supporting the hypothesis of trisynaptic pathway dysfunction in schizophrenia [2,38]. In non-schizophrenia subjects, the presence of newly generated neurons in the granule cell layer of the DG are thought to be a result of, or an indicator of neuroplasticity [39]. In contrast, similar neuroplastic-driven neuronal generation appeared suppressed in a postmortem study of schizophrenia [40]. Decreased volumes in DG may represent the lack of neurogenesis in schizophrenia.

Other studies of hippocampal subfields in schizophrenia using MRI also describe complementary findings to the present results. In a study of non-psychotic, first-degree relatives of patients with schizophrenia, the volume of the subiculum was smaller than controls with no family history of schizophrenia [18]. In patients with clinically stable, chronic schizophrenia, schizoaffective disorder, or bipolar disorder with psychosis, the CA2-3 subfield was most prominently affected, and an inverse correlation between subfield volumes and positive symptoms was noted [16]. In patients with approximately 8 years of illness, the severity of positive symptoms was inversely correlated with CA2-3 and CA1 subfield volumes [17]. Comparison of subfield volumes with healthy participants was not reported. Larger studies of patients with acute versus stable symptoms, and at first episode and chronic stages of illness will be needed before any definitive relationships between hippocampal subfield volumes and symptoms can be determined.

The CA4-DG subfield appeared sensitive to the effects of schizophrenia, as smaller volume was detected even at first episode. The increasing volume loss over time could relate to progression of schizophrenia [2]. Patients in our study were treated with antipsychotic drugs. Similar to the present observations, other investigators reported no statistically significant relationships between hippocampal subfield size or surface mapping and amount of antipsychotic drug treatment [12–14,17]. However, in other studies our group and others have reported that total hippocampal volumes and hippocampal shape may be sensitive to antipsychotic drug treatments, with some antipsychotics reducing volumes, and others ameliorating progressive, illness-related volume reduction [41,42]. The present study did not conclude the influence of antipsychotics on hippocampal volume and its subfield volume because duration and type of medication was not controlled. Further studies will be needed to investigate the possibility of antipsychotic drug contributions to subfield volume loss, and the possibility that non-pharmacological interventions such as aerobic exercise could ameliorate the progression of volume loss [43].

There are several limitations to our imaging approach. First, automated hippocampal subfield remains technically challenging. The Images we used were acquired with a 1.5T scanner, a strategy successfully applied in studies of Alzheimer’s and Parkinson’s diseases [19,28]. Although the mean subfield volumes we report in healthy volunteers and patients were similar to those reported in studies using 3T field strength [16,17], and demonstrated good reliability over time for healthy volunteers, higher resolution images would have been preferred. At this time, direct comparisons between hippocampal subfield volumes obtained at 1.5T and 3T are not available., Although higher field strength would theoretically improve image resolution and subsequently increase the accuracy of segmentation, this is not always the case due to the increased sensitivity to motion and magnetic susceptibility [44]. The image acquisition strategy used here was based on a T1 sequence, as in the original report of the subfield algorithm [15,17]. Other studies report advantages of T2 sequences [45,46], or combined high resolution T1 and T2 sequences to improve definition of hippocampal subfields [47]. The automatic segmentation approach was developed to apply manually delineated subfields from 3T images into a probablistic atlas or mask to be implemented across varying image sets. A direct comparison of manually delineated subfields and the automatic segmentation approach was not feasible in our sample set, and this is a limitation of the study. However, as stated in [12], “manual delineations suffer from intra- and interobserver variability, which confounds subsequent statistical analyses of the results." Despite these difficulties, the representative images obtained in our study appeared similar to those reported by other groups [15,17,19,28]. Second, the small sample size in each group might not be sufficient to detect subtle volume changes. The effects of laterality, sex, and medications were difficult to interpret in this context. Analysis of larger numbers of patients will be required to characterize detailed volumetric change and to control for potential effects of modifying factors. Third, we did not examine the possible influence of factors such as IQ, BMI, and psychological stress on hippocampal total or subfield volumes. Additional anatomical information such as the anterior and posterior segment volumes would also add value.

In summary, data from this study provides empirical evidence of effects of illness, and of illness progression on volumes of hippocampal subfields. As imaging techniques continue to improve, study of hippocampal subfields in schizophrenia may provide important insights into the dynamic characteristics of the illness and of neuroplasticity related to treatment.
